# Dezocine exhibits antihypersensitivity activities in neuropathy through spinal μ-opioid receptor activation and norepinephrine reuptake inhibition

**DOI:** 10.1038/srep43137

**Published:** 2017-02-23

**Authors:** Yong-Xiang Wang, Xiao-Fang Mao, Teng-Fei Li, Nian Gong, Ma-Zhong Zhang

**Affiliations:** 1 King’s Lab, Shanghai Jiao Tong University School of Pharmacy, 800 Dongchuan Road, Shanghai 200240, China; 2 Department of Anesthesiology and Pediatric Clinical Pharmacology Laboratory, Shanghai Children’s Medical Center, Shanghai Jiao Tong University School of Medicine, 1678 Dongfang Road, Shanghai 200127, China

## Abstract

Dezocine is the number one opioid painkiller prescribed and sold in China, occupying 44% of the nation’s opioid analgesics market today and far ahead of the gold-standard morphine. We discovered the mechanisms underlying dezocine antihypersensitivity activity and assessed their implications to antihypersensitivity tolerance. Dezocine, given subcutaneously in spinal nerve-ligated neuropathic rats, time- and dose-dependently produced mechanical antiallodynia and thermal antihyperalgesia, significantly increased ipsilateral spinal norepinephrine and serotonin levels, and induced less antiallodynic tolerance than morphine. Its mechanical antiallodynia was partially (40% or 60%) and completely (100%) attenuated by spinal μ-opioid receptor (MOR) antagonism or norepinephrine depletion/α_2_-adrenoceptor antagonism and combined antagonism of MORs and α_2_-adenoceptors, respectively. In contrast, antagonism of spinal κ-opioid receptors (KORs) and δ-opioid receptors (DORs) or depletion of spinal serotonin did not significantly alter dezocine antiallodynia. In addition, dezocine-delayed antiallodynic tolerance was accelerated by spinal norepinephrine depletion/α_2_-adenoceptor antagonism. Thus dezocine produces antihypersensitivity activity through spinal MOR activation and norepinephrine reuptake inhibition (NRI), but apparently not through spinal KOR and DOR activation, serotonin reuptake inhibition or other mechanisms. Our findings reclassify dezocine as the first analgesic of the recently proposed MOR-NRI, and reveal its potential as an alternative to as well as concurrent use with morphine in treating pain.

The centrally acting opioid analgesic dezocine represents one of the most miraculous pain medicines with respect to its utilization history and mechanisms of action. Dezocine was first discovered and developed by Wyeth-Ayerst (now Pfizer) and introduced to the market in 1990 to treat perioperative pain. It was voluntarily withdrawn from the market by the manufacturer in 2000 (http://www.fda.gov/ohrms/dockets/98fr/091300c.htm). However, dezocine in injection form was redeveloped by Yangzi River in China for the same indication in 2009, and quickly became heavily prescribed for perioperative pain (from intraoperative to ward analgesia) and injury pain. Recently, it has also gained acceptance to treat visceral and cancer pain, although there is limited information available for its use for neuropathic pain^1^,^2^,^3^,^4^. To date, it accounts for the number one opioid painkiller prescribed and sold in China, with 44% of the nation’s opioid analgesics market. This popularity is far ahead of the globally recognized gold-standard painkiller morphine, which has a market share of only 2% in China (PDB database, China Pharmaceutical Information Center, Shanghai, China). Such extensive clinical use may be attributed to its high analgesic efficacy, broad analgesic activity profile, favorable tolerability, and beneficial profile over morphine and other opioids used in clinical practice. Compared to morphine, dezocine has less liability of physical dependence^5^, analgesic tolerance^3^,^6^ and opioid-induced hyperalgesia^7^. Indeed, dezocine is not a controlled substance classified by the World Health Organization, and is listed in Category II of the psychotropic drugs in China and the United States^8^. There are no reports regarding addiction to dezocine^9^. Additional advantages over morphine and other opioids include less respiratory depression, better gastrointestinal tolerability, and less cough and itch^10^,^11^,^12^.

Dezocine was rationally designed to be the bridged aminotetralin analog of pentazocine to improve analgesic efficacy, the latter was developed in 1970s and claimed to be a mixed opioid receptor partial agonist/antagonist^5^,^13^. The chemical structures of pentazocine and dezocine are presented in Fig. 1. The structural similarity caused dezocine to be classified in the category of mixed opioid receptor partial agonist/antagonist as pentazocine, well documented in textbooks, regulatory guidelines, and literatures without exception. It has been generally believed that the beneficial profile of dezocine over morphine and other opioids is due to its opioid receptor partial agonist/antagonist property. In fact, dezocine has a bioactivity in interactions with the μ-opioid receptor (MOR), κ-opioid receptor (KOR), and δ-opioid receptor (DOR) with different binding affinities and activities, ranging from nM (MOR as the highest) to sub μM (DOR as the least)^8^,^14^,^15^,^16^. However, it is still unclear to date whether dezocine is a full or partial MOR agonist^8^,^15^,^17^,^18^, and whether dezocine is an agonist or antagonist of KORs^8^,^16^. In a recent study, dezocine was shown to inhibit norepinephrine and serotonin reuptake with _p_IC_50_ values of 5.68 and 5.86, but had apparently low affinity to the dopamine transporter^8^. To date, there are only limited controversial results on MORs and KORs reported that touched on the role of these potential target molecules, including monoamine transporters and opioid receptor subtypes, in dezocine analgesia in the experimental animals or humans^5^,^17^,^18^,^19^,^20^.

The great contrast between the popular clinic usage and limited pharmacological understanding prompted us to investigate the mechanistic rationales underlying dezocine analgesia. Methodologically, we employed a rat model of neuropathic pain induced by tight ligation of L5/L6 spinal nerves in this study. This model is widely accepted for the robust behavioral response, good reproduction, and close clinical relevance especially when the neuropathy is well established^21^. Our specific aims were 1) to test dezocine mechanical antiallodynia and thermal antihyperalgesia in neuropathy; 2) to illustrate the mediation of spinal opioid receptor subtype activation, spinal norepinephrine reuptake inhibition (NRI) and serotonin reuptake inhibition (SRI) in dezocine mechanical antiallodynia; and 3) to assess dezocine mechanical antiallodynic tolerance and its mechanism of action. For comparison, morphine was tested in parallel with dezocine throughout the study.

## Results

### Dezocine produced profound mechanical antiallodynia and thermal antihyperalgesia in neuropathy

**Figure 2:**
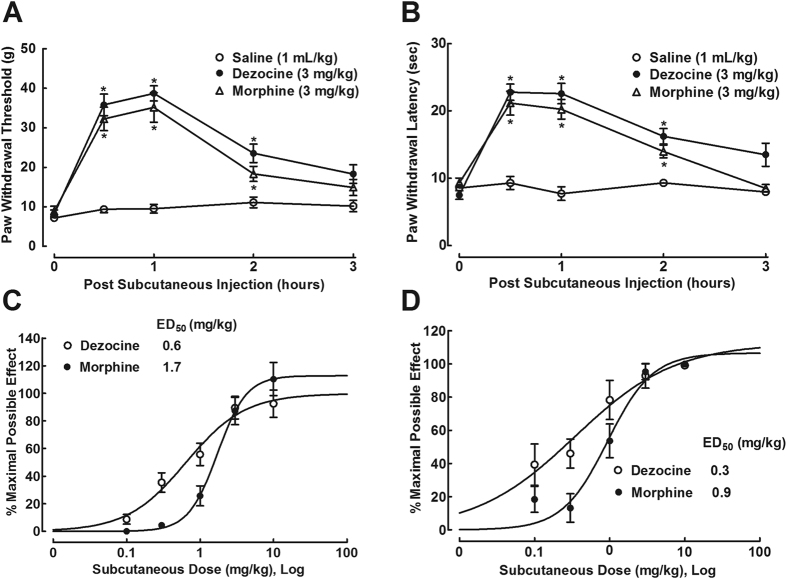
Time-course (**A**,**B**) and cumulative dose-response curves (**C–F**) of subcutaneous injection of dezocine and morphine antihypersensitivity activity in neuropathy. Neuropathic rats, induced by tight ligation of L5/L6 spinal nerves, received subcutaneous injection of dezocine or morphine. Data are summarized as means ± SEM (n = 6 and 10 in each group in the time-course and dose-response study, respectively). *P < 0.05 vs. the normal saline control, by repeats-measured two-way ANOVA followed by post-hoc student-Newman-Keuls tests.

The antihypersensitivity activity effects of dezocine and morphine were assessed in neuropathic rats one week after spinal nerve ligation. The baseline mechanical thresholds and thermal latencies in contralateral and ipsilateral paws were around 40 g and 6 g, and 14 and 8 sec, respectively. Three groups of neuropathic rats received subcutaneous normal saline (1 mL/kg), dezocine (3 mg/kg) or morphine (3 mg/kg). Withdrawal responses to mechanical and radiant heat stimuli were consecutively (with 10-min interval) measured in ipsilateral hindpaws prior to and 0.5, 1, 2, and 3 hours post injection. The mechanical thresholds in normal saline-injected rats remained unchanged during the observation period of time. However, both dezocine and morphine markedly inhibited mechanical thresholds. The mechanical antiallodynic effects of dezocine and morphine (with slightly shorter duration) were time-dependent and lasted for approximately 3 hours, peaked at 0.5–1 hour after injection (F_2;162_ = 52.6, P < 0.05 by repeats-measured two-way ANOVA followed by the post-hoc Student-Newman-Keuls test; Fig. 2A). Similarly, both subcutaneous dezocine and morphine prolonged thermal withdrawal latencies in ipsilateral hindpaws in a time-dependent manner (F_2;162_ = 44.13, P < 0.05 by repeats-measured two-way ANOVA followed by the post-hoc Student-Newman-Keuls test; Fig. 2B).

Cumulative dose (0.1, 0.3, 1, 3 and 10 mg/kg) responses of both drugs were conducted in neuropathic rats. Given subcutaneously, both dezocine and morphine dose-dependently inhibited mechanical thresholds in ipsilateral hindpaws. After transformation of mechanical thresholds to percentage of maximum possible effect (% MPE), dose-response analyses showed that their projected E_max_ values were 98% and 113% MPE, and ED_50_ values were 0.6 mg/kg (95% confidence: 0.3–1.3 mg/kg) and 1.7 mg/kg (95% confidence: 1.2–2.4 mg/kg), respectively (Fig. 2C). Similarly, both dezocine and morphine produced a dose-dependent inhibition of thermal withdrawal response, with E_max_ values of 112% MPE and 107% MPE, and ED_50_s of 0.3 mg/kg (95% confidence: 0.1–0.4 mg/kg) and 0.9 mg/kg (95% confidence: 0.6–1.2 mg/kg, P < 0.05 by two-tailed and unpaired Student t-test, compared to dezocine), respectively (Fig. 2D).

### Antagonism of spinal MORs partially attenuated dezocine mechanical antiallodynia

**Figure 3:**
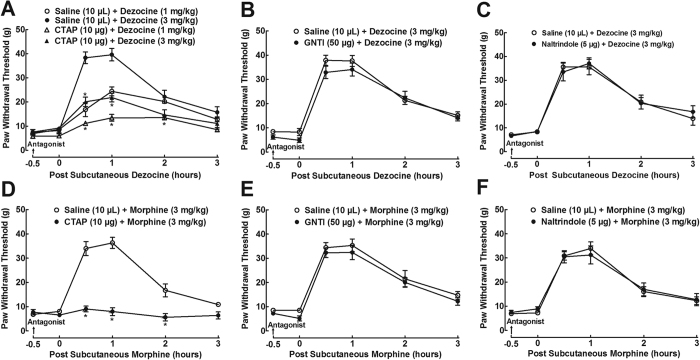
Effects of the specific opioid receptor subtype antagonists, given intrathecally, on dezocine (**A–C**) and morphine antinociception (**D–F**) in neuropathic rats, induced by tight ligation of L5/L6 spinal nerves. Data are presented as means ± SEM (n = 6–8 in each group). *P < 0.05 vs. the respective normal saline + dezocine and normal saline + morphine group, by repeats-measured two-way ANOVA followed by post-hoc student-Newman-Keuls tests.

Dezocine can interact with MORs, KORs and DORs with different binding affinities and activities^8^,^15^,^16^. To determine which subtype of the spinal receptors was involved in dezocine mechanical antiallodynia, the specific MOR antagonist CTAP, KOR antagonist 5′-guanidinonaltrindole (GNTI) and DOR antagonist naltrindole were employed in neuropathic rats. As shown in Fig. 3, treatment with intrathecal injection of CTAP (10 μg), GNTI (50 μg) and naltrindole (50 μg) did not significantly alter baseline mechanical thresholds in ipsilateral hindpaws, compared to normal saline (10 μL)-treated control rats. Subcutaneous injection of dezocine (1 and 3 mg/kg) produced time- and dose-dependent mechanical antiallodynia, which was partially prevented by the pretreatment (0.5 hours prior to) with intrathecal injection of CTAP, with a 63 ± 4% and 59 ± 6% inhibition, respectively (F_3;120_ = 47.2, P < 0.05 by repeats-measured two-way ANOVA followed by the post-hoc Student-Newman-Keuls test; Fig. 3A). In contrast, pretreatment with intrathecal GNTI (Fig. 3B) and naltrindole (Fig. 3C) failed to reduce dezocine mechanical antiallodynia. On the other hand, subcutaneous injection of morphine (3 mg/kg) also caused a time-dependent mechanical antiallodynia, which was completely blocked by intrathecal pre-injection of CTAP (F_1;60_ = 86.6, P < 0.05 by repeats-measured two-way ANOVA followed by the post-hoc Student-Newman-Keuls test; Fig. 3D). Conversely, intrathecal pretreatment with GNTI (Fig. 3E) and naltrindole (Fig. 3F) did not significantly inhibit morphine mechanical antiallodynia.

### Depletion of spinal norepinephrine partially attenuated dezocine mechanical antiallodynia

**Figure 4:**
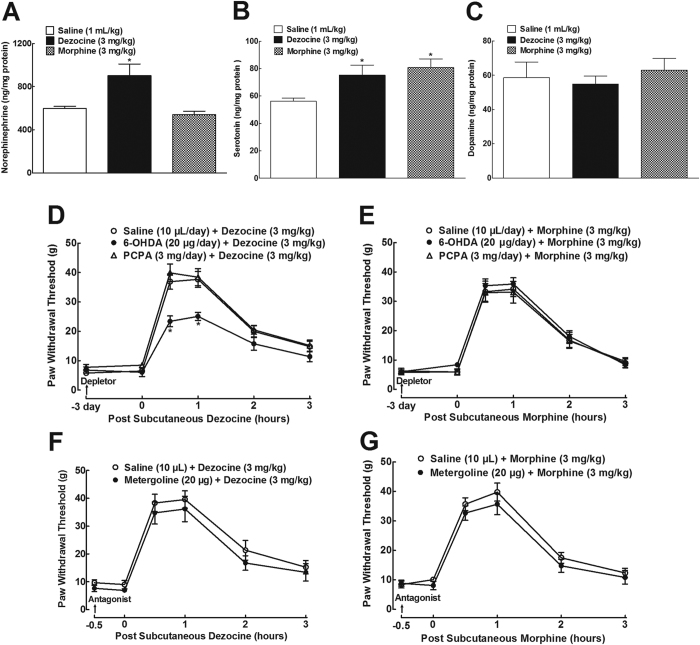
Effects of dezocine and morphine, given subcutaneously, on spinal levels of norepinephrine (**A**), serotonin (**B**) and dopamine (**C**) in neuropathic rats, induced by tight ligation of L5/L6 spinal nerves. Effects of the specific norepinephrine depletor 6-hydroxydopamine (6-OHDA), serotonin depletor *p*-cholrophenylalanine (PCPA) (**D, E**) and 5-hydroxytryptamine (5-HT) receptor antagonist metergoline (**F**,**G**), given intrathecally, on dezocine and morphine mechanical antiallodynia in neuropathy. Data are presented as means ± SEM (n = 6 in each group). *P < 0.05 vs. the normal saline control or respective normal saline + dezocine and normal saline + morphine group, by one-way or repeats-measured two-way ANOVA followed by post-hoc student-Newman-Keuls tests.

Dezocine was reported to inhibit norepinephrine and serotonin reuptake in HEK293 cells stably expressed the individual norepinephrine and serotonin transporter^8^. To confirm its blockade effects on norepinephrine and serotonin reuptake in the spinal cord, the spinal levels of norepinephrine, serotonin, and dopamine were measured after administration of dezocine. Three groups of neuropathic rats received subcutaneous normal saline (1 mL/kg), dezocine (3 mg/kg) or morphine (3 mg/kg), and sacrificed 1 hour later to isolate the ipsilateral spinal lumbar segments. The levels of norepinephrine, serotonin, and dopamine in the spinal homogenates were measured using commercial enzyme-linked immunosorbent assay (ELISA) kits. The spinal baseline levels of norepinephrine, serotonin and dopamine were 600 ± 21, 56.1 ± 2.4 and 58.6 ± 9.2 ng/mg protein, respectively. As shown in Fig. 4A–C, although subcutaneous dezocine did not significantly affect the dopamine level, it significantly increased the norepinephrine and serotonin levels by 34% and 25%, respectively (F = 8.557, *P* < 0.05 by one-way ANOVA followed by the post-hoc Student-Newman-Keuls test). On the other hand, subcutaneous morphine significantly increased spinal serotonin levels by 30% (F = 5.197, *P* < 0.05 by one-way ANOVA followed by the post-hoc Student-Newman-Keuls test), without altering the spinal levels of norepinephrine and dopamine.

Spinal norepinephrine and serotonin have been involved in pain transmission and process to a certain extent. We thus assessed the contribution of the increased spinal norepinephrine and serotonin to dezocine antinociception by employing specific norepinephrine depletor 6-hydroxydopamine (6-OHDA) and serotonin depletor *p*-cholrophenylalanine (PCPA). As shown in Fig. 4D,E, 3-day intrathecal injections of 6-OHDA (20 μg/day) and PCPA (3 mg/day) did not significantly alter mechanical withdrawal thresholds in ipsilateral paws, compared to normal saline (10 μL/day)-treated control rats. On the fourth day, a single bolus subcutaneous injection of dezocine (3 mg/kg) in saline-treated rats produced a marked mechanical antiallodynia, which was partially reduced by pretreatment of 6-OHDA with an inhibitory rate of 37 ± 4% (F_2;90_ = 7.993, *P* < 0.05 by repeats-measured two-way ANOVA followed by the post-hoc Student-Newman-Keuls test). In contrast, pretreatment with PCPA over 3 days did not significantly alter dezocine mechanical antiallodynia (Fig. 4D). In comparison, both 6-OHDA and PCPA did not significantly alter morphine (3 mg/kg) mechanical antiallodynia (Fig. 4E).

To further explore the disassociation between spinal serotonin and dezocine antinociception, the nonselective 5-hydroxytryptamine (5-HT) receptor antagonist metergoline (20 μg) was employed. Intrathecal metergoline did not significantly affect baseline mechanical thresholds in ipsilateral hindpaws, or dezocine (3 mg/kg, Fig. 4F) and morphine (3 mg/kg) mechanical antiallodynia (Fig. 4G).

### Antagonism of spinal noradrenergic α_2_-adrenoceptors partially attenuated dezocine mechanical antiallodynia

**Figure 5:**
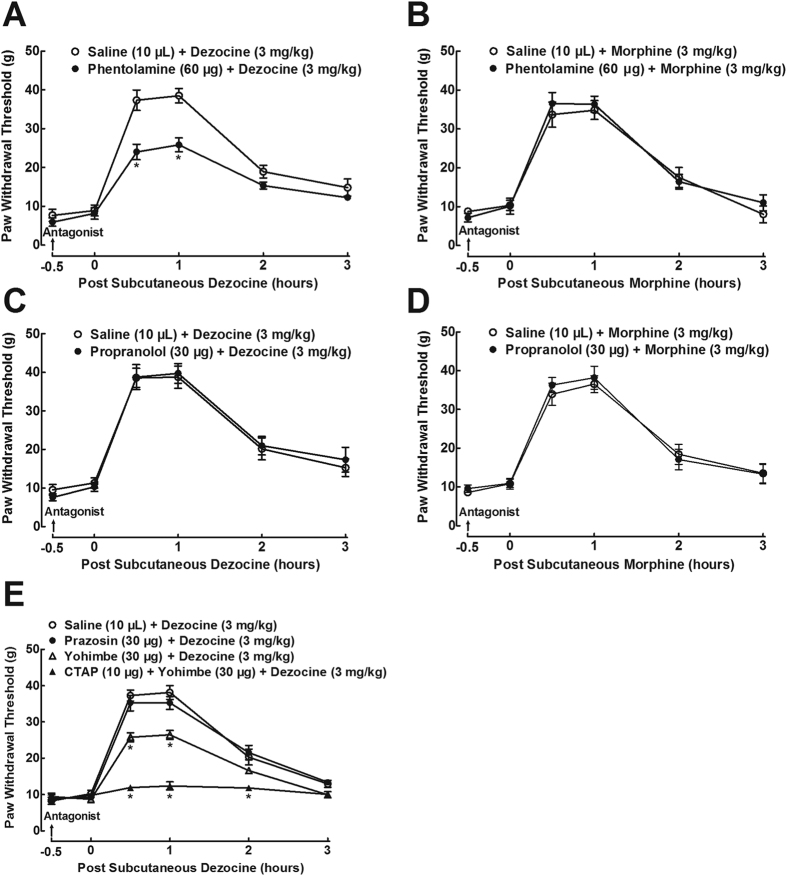
Effects of the selective noradrenergic α-adrenoceptor antagonist phentolamine (**A**,**B**), β-adrenoceptor antagonist propranolol (**C**,**D**), α_1_-adrenoceptor antagonist prazosin, α_2_-adrenoceptor receptor antagonist yohimbe, and the mixture of the μ-opioid receptor antagonist CTAP and yohimbe (**E**), given intrathecally, on dezocine and morphine antinociception in neuropathic rats, induced by tight ligation of L5/L6 spinal nerves. Data are presented as means ± SEM (n = 6 in each group). *P < 0.05 vs. the normal saline control or respective normal saline + dezocine and normal saline + morphine group, by repeats-measured two-way ANOVA followed by post-hoc student-Newman-Keuls tests.

Since increase in spinal norepinephrine is known to activate noradrenergic adrenoceptors to produce analgesia, we determined which subtypes of noradrenergic adrenoceptors were responsible for dezocine antinociception. As shown in Fig. 5A, intrathecal injection of the noradrenergic α-adrenoceptor antagonist phentolamine (60 μg) did not significantly affect mechanical thresholds in ipsilateral hindpaws, but its pretreatment (0.5 hours prior to) partially reduced dezocine (3 mg/kg) mechanical antiallodynia with an inhibitory rate of 36 ± 5% (F_1;60_ = 27.2, *P* < 0.05 by repeats-measured two-way ANOVA followed by the post-hoc Student-Newman-Keuls test). In contrast, intrathecal phentolamine did not significantly affect morphine (3 mg/kg) mechanical antiallodynia (Fig. 5B). On the other hand, intrathecal injection of the noradrenergic β-adrenoceptor antagonist propranolol (30 μg) did not significantly alter baseline mechanical thresholds in ipsilateral hindpaws or dezocine (Fig. 5C) and morphine (Fig. 5D) mechanical antiallodynia.

To further identify the subtypes of α-adrenoceptors associated with dezocine antinociception, the specific noradrenergic α_1_- and α_2_-adrenoceptor antagonists prazosin and yohimbe were used. As exhibited in Fig. 5E, intrathecal injection of yohimbe (30 μg) did not significantly affect baseline mechanical thresholds in ipsilateral hindpaws, but its pretreatment (0.5 hours earlier) partially attenuated dezocine (3 mg/kg) mechanical antiallodynia with a 35 ± 4% inhibition (F_3;120_ = 29.7, *P* < 0.05 by repeats-measured two-way ANOVA followed by the post-hoc Student-Newman-Keuls test). In contrast, intrathecal prazosin (30 μg) did not significantly alter baseline mechanical thresholds in ipsilateral hindpaws or dezocine mechanical antiallodynia.

More excitingly, although intrathecal injection of the mixture of yohimbe (30 μg) and CTAP (10 μg) did not significantly alter baseline mechanical thresholds in ipsilateral paws, its pretreatment (0.5 hours prior to) completely blocked dezocine mechanical antiallodynia with a 92 ± 4% inhibition (F_3;120_ = 29.7, *P* < 0.05 by repeats-measured two-way ANOVA followed by the post-hoc Student-Newman-Keuls test).

### Spinal norepinephrine depletion and noradrenergic α_2_-adrenoceptor antagonism accelerated dezocine antiallodynic tolerance

**Figure 6:**
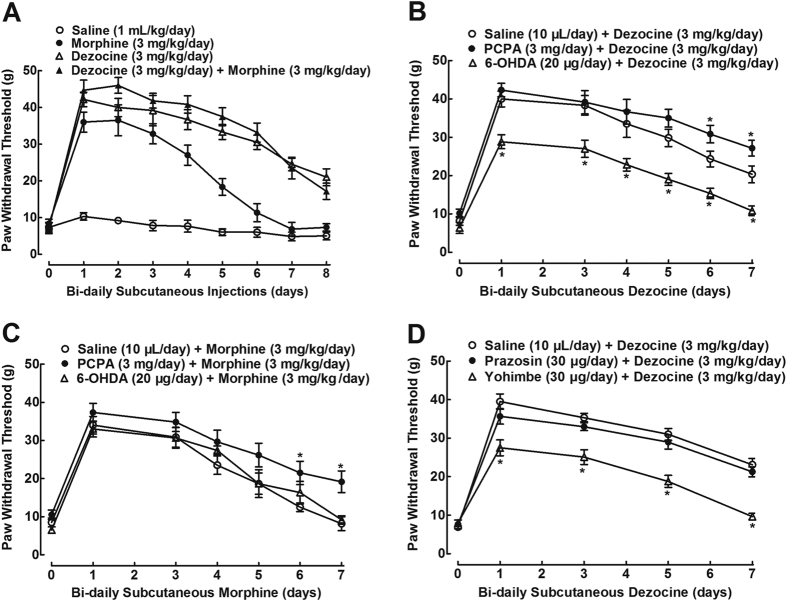
Mechanical antiallodynic tolerance development of dezocine, morphine and their combination, given subcutaneously, in neuropathic rats, induced by tight ligation of L5/L6 spinal nerves (**A**). Effects of the specific norepinephrine depletor 6-hydroxydopamine (6-OHDA), serotonin depletor *p*-cholrophenylalanine (PCPA) (**B**,**C**), and the selective noradrenergic α_1_-adrenoceptor antagonist prazosin and α_2_-adrenoceptor receptor antagonist yohimbe (**D**), given intrathecally, on dezocine and morphine antinociceptive tolerance in neuropathy. Data are presented as means ± SEM (n = 6 in each group). *P < 0.05 vs. the normal saline control or respective normal saline + 6-OHDA and normal saline + PCPA group, by repeats-measured two-way ANOVA followed by post-hoc student-Newman-Keuls tests.

We then examined dezocine antiallodynic tolerance. Four groups of neuropathic rats received multiple subcutaneous injections of normal saline (1 mL/kg), dezocine (3 mg/kg), morphine (3 mg/kg) and their combination twice daily (9:00 am and 9:00 pm) for 8 consecutive days. Withdrawal thresholds in ipsilateral hindpaws were measured 1 hour after each morning injection. As shown in Fig. 6A, mechanical thresholds in the normal saline control rats remained unchanged during the period of observation. The first subcutaneous morphine injection produced marked mechanical antiallodynia in ipsilateral paws. Its continued injections induced progressive and complete mechanical antiallodynic tolerance, with a biological T_1/2_ of 4.3 ± 0.5 days. Similar to morphine, single subcutaneous dezocine produced marked mechanical antiallodynia but its continued injections also induced antiallodynic tolerance. However, dezocine developed antiallodynic tolerance much more slowly than morphine, with a biological T_1/2_ of 6.9 ± 0.3 days, which was statistical significantly larger than that of morphine (F = 163.9, P < 0.05 by one-way ANOVA followed by the post-hoc Student-Newman-Keuls test). Concurrent single injection of dezocine with morphine produced apparently additive mechanical antiallodynia, but their bi-daily concurrent injections induced the same mechanical antiallodynic tolerance as dezocine alone, with a biological T_1/2_ of 6.8 ± 0.5 days, which was statistical significantly larger than that of morphine (F = 163.9, P < 0.05 by one-way ANOVA followed by the post-hoc Student-Newman-Keuls test).

To assess the contribution of the increased spinal norepinephrine and serotonin levels to dezocine-associated antinociceptive tolerance, 6-OHDA and PCPA were employed. Multiple bi-daily subcutaneous injections of dezocine (3 mg/kg/day, bidaily) produced slow development of antiallodynic tolerance with a biological T_1/2_ of 6.7 ± 0.4 days. Intrathecal pre-injections of 6-OHDA (20 μg/day) for 3 days significantly reduced dezocine mechanical antiallodynia by a nearly fixed magnitude over the period of repeat administration (F_2;105_ = 13.6, P < 0.05 by repeats-measured two-way ANOVA followed by the post-hoc Student-Newman-Keuls test), with a reduced biological T_1/2_ of 5.1 ± 0.4 days for dezocine. In contrast, intrathecal pre-injections of PCPA (3 mg/day) for 3 days significantly delayed dezocine antiallodynic tolerance (F_2;105_ = 13.6, P < 0.05 by repeats-measured two-way ANOVA followed by the post-hoc Student-Newman-Keuls test), with an increased biological T_1/2_ of 7.4 ± 0.2 days for dezocine (Fig. 6B).

In comparison, multiple bi-daily subcutaneous morphine (3 mg/kg/day) injections produced progressive and complete mechanical antiallodynic tolerance with a biological T_1/2_ of 4.7 ± 0.3 days. Intrathecal pre-injections of 6-OHDA for 3 days did not significantly alter morphine mechanical antiallodynic tolerance, with a biological T_1/2_ of 5.2 ± 0.3 days for morphine. In contrast, intrathecal pre-injections of PCPA for 3 days significantly delayed morphine mechanical antiallodynic tolerance (F_2;105_ = 2.9, P < 0.05 by repeats-measured two-way ANOVA followed by the post-hoc Student-Newman-Keuls test), with an increased biological T_1/2_ of 5.6 ± 0.2 days for morphine (Fig. 6C).

To further determine whether spinal noradrenergic α_2_-adrenoceptors contributed to dezocine-delayed antinociceptive tolerance, prazosin and yohimbe were employed. As shown in Fig. 6D, bi-daily dezocine induced a delayed mechanical antiallodynic tolerance with a biological T_1/2_ of 6.4 ± 0.4 days. Bi-daily intrathecal concurrent injections of yohimbe significantly reduced dezocine mechanical antiallodynia by a nearly fixed magnitude over the period of repeat administrations (F_2;75_ = 30.6, P < 0.05 by repeats-measured two-way ANOVA followed by the post-hoc Student-Newman-Keuls test), with a reduced biological T_1/2_ of 5.0 ± 0.3 days for dezocine. In contrast, intrathecal prazosin did not significantly affect dezocine antiallodynic tolerance, with a biological T_1/2_ of 6.3 ± 0.3 days for dezocine.

## Discussion

In this study, we have for the first time, demonstrated that dezocine antihypersensitivity activity in neuropathy is due to spinal MOR activation and NRI, but apparently not due to other pharmacological mechanisms especially spinal KOR and DOR activation or SRI. This newly reached conclusion is consistently supported by the summarized findings below.

First, dezocine was a potent agonist of MORs with the potency in a range of nM, slightly higher than that of morphine^8^,^15^. Its efficacy was also close to that of the full agonist morphine, but lower than other higher efficacy opioid opioids, such as fentanyl, hydromorphone, and β-endorphin, leading to its traditional classification of partial agonist^8^,^15^. In this study, intrathecal injection of the specific MOR antagonist CTAP majorly (approximately 60%) inhibited subcutaneous dezocine (1 and 3 mg/kg)-induced mechanical antiallodynia in neuropathy, consistent with the reported MOR antagonists naltrexone and β-funaltrexamine that attenuated dezocine antinociception in the rat tail-withdrawal assay^17^,^18^, although high doses of naloxone and β-funaltrexamine were unable to antagonize its rate decreasing effect in pigeons^19^. On the contrary, intrathecal the specific DOR antagonist naltrindole failed to attenuate dezocine mechanical antiallodynia. It is not surprising to observe the inability of naltrindole, as dezocine has the least affinity (in sub μM range) to DORs among all opioid receptors^8^,^15^,^16^. On the other hand, dezocine was initially assumed to be a KOR agonist, due to its structural similarity to pentazocine that was claimed to have KOR agonist activity^8^,^19^. A domestic report claimed that intraperitoneal dezocine-induced antinociception was completely attenuated by another KOR antagonist nor-BNI in the mouse thermally acute reflex nociception and acetic acid-induced writhing response^20^. However, dezocine was recently demonstrated to be a KOR antagonist (rather than an agonist) with a potency of roughly 30 nM, measured by a G-protein activation assay in mammalian cells exclusively expressed KORs^8^. In our study, GNTI at an effective dose did not affect either baseline pain thresholds or dezocine mechanical antiallodynia, suggesting that dezocine antinociception is not correlated with activation of spinal KORs. While the reason of this discrepancy with Qiao *et al*.^20^ is unclear, nor-BNI was found to be an inverse agonist of KORs^8^,^22^, and its inverse agonism may confound dezocine antinociception and the claimed conclusion may not be necessarily reached. In comparison, CTAP completely blocked morphine mechanical antiallodynia, while GNTI and naltrindole lacked such a blockade effect, although morphine is well-known to interact with all of opioid receptor subtypes with different affinities^8^,^14^,^15^,^16^,^23^. Morphine-induced antinociception was hence simply a pure spinal MOR activation, consistent with a recent report using MOR gene knockout mice^24^.

Furthermore, dezocine was recently demonstrated to be a NRI and SRI, by occupying the binding site of known NRIs and SRIs predicted by a computational docking calculation, with a reasonably high potency in a range of μM^8^. The current study demonstrated that dezocine significantly increased ipsilateral spinal levels of norepinephrine, but not of dopamine. Selectively, 3-day intrathecal 6-OHDA specifically depleted spinal norepinephrine^25^ and minorly (approximately 40%) reduced dezocine (but not morphine) mechanical antiallodynia. Increased norepinephrine in the synapse leads to activation of adrenoceptors^26^,^27^, and truly in our study, intrathecal injection of the nonselective α-adrenoceptor antagonist phentolamine and selective α_2_-adrenoceptor antagonist yohimbe minorly (approximately 40%) attenuated dezocine mechanical antiallodynia, whereas the non-selective β-adrenoceptor antagonist propranolol and selective α_1_-adrenoceptor antagonist prazosin failed to have such an attenuation. In contrast, phentolamine and yohimbe did not attenuate morphine mechanical antiallodynia. On the other hand, subcutaneous dezocine also increased spinal serotonin levels. However, depletion of spinal serotonin by PCPA^28^ and blockade of 5-HT receptors by metergoline^29^,^30^ failed to affect dezocine mechanical antiallodynia. Although morphine also increased spinal serotonin (but not norepinephrine) levels as reported previously^31^, intrathecal injection of PCPA and metergoline did not alter morphine mechanical antiallodynia, consistent with the previous data^32^. Thus, increase in spinal serotonin via reuptake inhibition does not seem to contribute to dezocine and morphine mechanical antiallodynia. Although our notion goes along with the increasingly accepted concept that the role of spinal serotonin in the context of pain therapy is less clear and the serotonin transporter plays a far smaller role in pain and analgesia than the norepinephrine transporter^33^,^34^,^35^,^36^, it does not exclude the possibility that SRI may contribute to dezocine-induced other biological functions, such as potential inhibition of comorbid depression.

Finally, combination of intrathecal CTAP and yohimbe completely (up to 100%) blocked dezocine antinociception, which clearly indicates that both direct activation of spinal MORs and indirect activation of noradrenergic α_2_-adrenoceptors via NRI fully account for dezocine mechanical antiallodynia, and there are seemingly no other pharmacological mechanisms involved. The successful story of dezocine in controlling perioperative pain is helpful to validate an effective therapeutic approach. Spinal norepinephrine plays an important role in pain transmission and transduction, including potentiation of μ-opioid activity^37^, and NRIs have been used for decades in treatment of chronic pain^38^,^39^. Because of the synergistic analgesic effect, even relatively moderate activity in the two target sites (μM in NRI for dezocine) within a given chemical molecule may be sufficient to produce strong analgesia. Thus, simultaneous interaction with both MORs and norepinephrine transporters residing in one single molecule with continuously fixed contribution (60% vs. 40% for dezocine) would expect to generate efficient analgesics with an improved tolerability profile. Such a concept, fortunately, had been taken by the pharmaceutical industry and a novel category of MOR-NRI has been recently proposed^40^,^41^. Tapentadol is a MOR-NRI with minimal serotonin activities, approved seven years ago to treat moderate to severe acute and chronic pain in adults, available in the immediate release form for acute pain and the extended-release formulation for chronic pain. Its high efficacy has been demonstrated in a variety of animal models of nociceptive, inflammatory, and chronic neuropathic pain as well as in clinical studies with pains arising from different etiologies. Advantages over morphine regarding gastrointestinal tolerability, tolerance development, physical dependence, and low level of abuse and diversion were shown in animal studies and human data, although it is still listed as a controlled substance^41^,^42^,^43^. Tapentadol has been suggested to be the first representative of the proposed new class of MOR-NRI^40^,^41^,^43^. However, dezocine would have been the first analgesic in this class given that it has been in clinical use for 26 years, although its clear dual mechanism is just being illustrated today.

Dezocine has been shown to have less liability of analgesic tolerance^3^,^6^ and physical dependence over morphine and other opioids, although the literature regarding dezocine antinociceptive tolerance in experimental animals is somewhat controversial. A previous study, conducted two decades ago, demonstrated that intrathecal dezocine in rats developed a slow but complete antinociceptive tolerance in thermally evoked nociceptive pain in two weeks^6^. In contrast, a recent study reported that bi-daily intraperitoneal dezocine over 10 consecutive days did not produce any tolerance in neuropathic pain, which was induced by chronic constriction injury of sciatic nerve^3^. We performed the tolerance experiment in spinal nerve ligation-induced neuropathic pain and employed morphine as a positive control. Bi-daily dezocine produced a delayed antiallodynic tolerance compared to morphine. Further mechanistic study demonstrated that PCPA-induced spinal serotonin depletion significantly restored chronic dezocine-reduced mechanical antiallodynic response, although it did not significantly affect dezocine mechanical antiallodynia after single injection. The potentiation is likely due to SRI on MOR activity, because PCPA as reported previously^27^,^44^ also potentiated chronic morphine-reduced mechanical antiallodynia, that had been demonstrated to be purely derived from MOR activation. In contrast, spinal norepinephrine depletion and noradrenergic α_2-_adrenoceptor antagonism (but not α_1_-adrenoceptor antagonism) significantly reduced dezocine (but not morphine) mechanical antiallodynia by a nearly fixed magnitude over the entire period of administrations, suggesting that dezocine induced less tolerance through its NRI-mediated potentiation of the reduced MOR activity following continuous exposure. The notion is supported by previous findings that NRIs and α_2_-adenoceptor agonists induced no antinociceptive tolerance and restored morphine antinociceptive responses^45^,^46^. The unique characterization of the less liability of tolerance and physical dependence makes dezocine an attractive alternative to or concurrent treatment with morphine to control severe chronic pain when continuous analgesia is required as the NRI property potentiates morphine analgesia leading to a reduction of “opioid load” and to no potentiation of its side-effects. Dezocine currently dominates the Chinese national opioid painkiller market by 44%, probably associated with the decreased portion (2%) of morphine (PDB database, China Pharmaceutical Information Center, Shanghai, China) and the much less trend of opioid epidemic in China. Treatment with morphine and other strong opioid analgesics is associated with serious adverse effects, such as constipation, nausea/vomiting, somnolence, respiratory depression, significantly decreasing the quality of life of patients, and even causing death, which occurs in thousands of cases each year in the USA^47^.

The other feature of the dual mechanisms is related to treating neuropathic pain, which is a debilitating symptom/disease and can be induced by numerous diseases, such as diabetes, cancer chemotherapy, surgical or accidental nerve trauma, or viral infection. Although etiologies are different, neuropathic pain is all related to nerve injury and principally involves the *N*-methyl-*D*-aspartate receptor-dependent central sensitization, disruption of descending inhibition, and glia activation^48^. The unique noradrenergic input and its combination with the opioid mechanism have a particular advantage in treatment of neuropathic pain^37^,^38^,^39^,^41^,^43^ and NRIs have been used in treatment of chronic pain. Indeed, a single subcutaneous dezocine produced pronounced mechanical antiallodynia and thermal antihyperalgesia in neuropathy, with duration slightly longer and potency slightly higher than morphine. Our finding is supported by the recent study that dezocine was effective in blockage of mechanical allodynia and thermal hyperalgesia in chronic constriction injury of sciatic nerve-induced neuropathic rats^3^. Thus the broad spectrum of analgesic activity of dezocine is potentially useful in control of neuropathic pain and other chronic pain including cancer pain. Future investigation would be helpful to differentiate pain pharmacology and clinical usage of dezocine and tapentadol by head to head comparison. Tapentadol is known to be less potent than dezocine in antinociception but orally available^43^.

## Materials and Methods

### Chemicals and reagents

Dezocine was a gift from Yangzi River Pharmaceuticals Group (Taizhou, Jiangsu, China). Morphine hydrochloride, prazosin hydrochloride, and yohimbe hydrochloride were obtained from the First Shenyang Pharmaceuticals (Shenyang, China) and metergoline and phentolamine mesilate were purchased from Tocris Bioscience (Bristol, UK) and Dalian Meilun Biology Technology Ltd. Co. (Dalian, China), respectively. CTAP, GNTI, naltrindole, PCPA methylester hydrochloride, and 6-OHDA hydrobromide were from Sigma-Aldrich (St. Louis, MO, USA). Dezocine was dissolved in normal saline and adjusted the pH to approximately 6.5 close to normal saline using the 1 N HCl solution, while CTAP, GNTI, naltrindole, propranolol, PCPA, and 6-OHDA were dissolved in normal saline.

### Animal experiments

Male adult (160–180 g) Wistar rats were purchased from the Shanghai Experimental Animal Institute (Shanghai, China) and kept in a temperature and humidity-controlled environment with a 12-hr light/dark cycle and *ad libitum* access to food and water. After adaptation of 3–5 days, rats underwent surgery for spinal nerve ligation and intrathecal catheterization. The research protocols were approved by the Animal Care and Welfare Committee of Shanghai Jiao Tong University and carried out in accordance with the animal care guidelines of the National Institutes of Health.

### Intrathecal catheterization and injection

Rats were anesthetized under inhalation of isoflurane 4% for induction and 1.5% for maintenance using an anesthesiameter (Ugo Basile Gas Anesthesia System, Comerio, Italy). Intrathecal catheterization and injection were performed as described previously^49^. Particularly, an 18-cm polyethylene catheter (PE-10: 0.28 mm i.d. and 0.61 mm o.d., Clay Adams, Parsippany, NJ) was inserted into the rat subarachnoid with approximate to the lumbar spinal cord and subcutaneously tunneled to the neck and fixed to the fascia. The intrathecal catheterization was verified 3 days after surgery by injecting 10 μl of 4% lidocaine, followed by 15 μl of normal saline flushing. Only rats that had no motor impairment following insertion of the intrathecal catheter and that developed immediate bilateral paralysis of the hindlimbs following intrathecal administration of lidocaine were selected for experiments.

### Spinal nerve ligation surgery

The rat spinal nerve ligation surgery was performed in accordance with previous studies^21^,^50^,^51^. In brief, after isoflurane anesthesia, the low back hairs were shaved, the skin was dissected by a surgical blade, and the paravertebral muscle was dissociated. As soon as the lumbar transverse process of L6 was carefully removed, the left L5 and L6 spinal nerves were isolated and tightly ligated with 6–0 silk suture. After ligation, the lumbar fascia was closed by 4–0 resorbable suture in layers and the skin was dusted with penicillin powder and sutured. The rats were returned to the housing cage for recovery. If intrathecal injection was needed, intrathecal catheterization was performed at the same time just before spinal nerve ligation. Only rats with marked ipsilateral allodynia to mechanical stimuli (hindpaw withdrawal thresholds in the operated side less than 10 g) and with no major motor impairment were included for experiments. The rats then were randomly allocated into each group (n = 6–10 per group as indicated in the figure legends) 1–2 weeks after surgery for pain behavioral test, in which the investigators were blind to the group assignments.

### Behavioral test of mechanical allodynia and heat hyperalgesia

The hindpaw withdrawal threshold to mechanical stimuli was measured using a 2290CE electrical von Frey hair (IITC Life Science Inc., CA, USA) according to previous studies^50^,^52^. Neuropathic rats were kept in plexiglass boxes (14 cm × 12 cm × 24 cm), which were placed on a mesh frame 40 cm above the table. The increasing force (ranging from 0.1 to 90 g) by an electronic monofilament was applied to the footpad to induce a sudden withdrawal response. Both hindpaws were measured subsequently and the lowest withdrawal producing force was recorded as a threshold, and each threshold was averaged from three-repeated measures at intervals of approximately 3 minutes each.

Thermal hindpaw withdrawal latency was measured according to a previous study^51^. Rats were placed in plexiglass boxes on elevated glass plate and, after 15 to 20 minute adaptation, the withdrawal latency was measured by turning on a radiant heat emitted by a 390G Plantar Test Analgesia Meter (IITC Life Science Inc.). The cut-off was set at 30 seconds to prevent tissue injury. The latency was recorded as the time from heat beam light turning on to hindpaw withdrawal. Both hindpaws were assessed subsequently three times with a 3-min interval. The value was averaged from the three repeated measurements.

In order to assess the blockade effects of a variety of antagonists and depletors on dezocine and morphine antinociception and antinociceptive tolerance, the following protocols were undertaken.

1. Antagonists for antinociception and antinociceptive tolerance: For the antinociception studies, neuropathic rats in each study received two treatments: single intrathecal injection of normal saline (10 μL) or antagonists 30 minutes followed by a subcutaneous injection of dezocine (3 mg/kg) or morphine (3 mg/kg). Withdrawal thresholds in response to mechanical stimuli in ipsilateral hindpaws were measured pre and post-injection (0, 0.5, 1, 2, and 3 hours). The time and dose regimens of the antagonists in the different studies were based on the following ref. 1) the selective MOR antagonist CTAP (10 μg), KOR antagonist GNTI (50 μg) and DOR antagonist naltrindole (5 μg)^52^,^53^; 2) nonselective 5-HT receptor antagonist metergoline (20 μg)^29^,^30^; 3) noradrenergic α-adrenoceptor antagonist phentolamine (60 μg) and β-adrenoceptor antagonist propranolol (30 μg)^54^,^55^; 4) selective noradrenergic α_1_-adrenoceptor antagonist prazosin (30 μg)^54^, noradrenergic α_2_-adrenoceptor antagonist yohimbe (30 μg)^38^, and the mixture of CTAP (10 μg) and yohimbe (30 μg). For the antinociceptive tolerance study, multiple bi-daily intrathecal injections of normal saline (10 μL), prazosin (30 μg) or yohimbe (30 μg), 30 minutes followed by multiple bi-daily subcutaneous injections of dezocine (3 mg/kg) for 7 days. Mechanical thresholds in ipsilateral hindpaws were measured 1 hour after the morning subcutaneous injection.
2. Depletors for antinociception and antinociceptive tolerance: For the antinociceptive study, neuropathic rats received two injections: daily intrathecal injections of normal saline (10 μL), 6-OHDA (20 μg) or PCPA (3 mg) for 3 days, followed by a subcutaneous injection of dezocine (3 mg/kg) or morphine (3 mg/kg) on the fourth day. The dose and time regimens of 6-OHDA and PCPA were based on previous studies in which they specifically depleted spinal and brain norepinephrine by approximately 40%^25^ and serotonin by approximately 65%^28^. Withdrawal thresholds in response to mechanical stimuli were measured in ipsilateral hindpaws pre- and post-injection (0, 0.5, 1, 2, and 3 hours). For the antinociceptive tolerance study, neuropathic rats received two treatments: daily intrathecal injections of normal saline (10 μL), 6-OHDA (20 μg) or PCPA (3 mg) for 3 days, followed by multiple bi-daily subcutaneous injections of dezocine (3 mg/kg) or morphine (3 mg/kg) on the fourth day for an additional 7 days. Mechanical thresholds in ipsilateral hindpaws were measured 1 hour after the morning subcutaneous injection.

### ELISA measurement of monoamines

To measure the spinal levels of norepinephrine, serotonin and dopamine, neuropathic rats were decapitated 1 hour post injection of dezocine or morphine, and ipsilateral spinal cords were collected and homogenated and centrifuged at 2,000 rpm/min for 5 minutes. The supernatant was collected and subjected to ELISA according to the manufacturer’s instructions (Nanjing Jiancheng Biotech, Nanjing, China). The monoamine levels were expressed as a relative ratio to the protein in the supernatant.

### Data evaluation and statistical analyses

% MPE was calculated using the formula: (post-drug mechanical threshold or thermal latency in ipsilateral hindlimb–baseline mechanical threshold or thermal latency in ipsilateral hindlimb)/(baseline mechanical threshold or thermal latency in contralateral hindlimb–baseline mechanical threshold or thermal latency in ipsilateral hindlimb) × 100^56^. The % MPE values closed to 100 indicate normal mechanical thresholds or thermal latency (i.e., near contralateral responses), while values closed to 0 indicate mechanical allodynia or thermal hyperalgesia. To calculate the inhibitory rate, the area under the curve between 0–3 hr (AUC_1–3 hr_) was calculated for the time-course of subcutaneous dezocine and morphine antinociception. The inhibitory rate (%) was used the following formula: 1–(Control AUC_1-3 hr_–Treatment AUC_1-3 hr_)/(Control AUC_1-3 hr_–Treatment AUC_1-3 hr_). The biological T_1/2_ for mechanical antiallodynia was calculated at the time between the durations when the antinociceptive effect declined to 50% of its peak value.

For the dose-response curve analysis, half-effective dose (ED_50_) were calculated by fitting nonlinear least-squares curves to the relation Y = a + bx, where x = [D]^*n*^/(ED50^*n*^ + [D]^*n*^). The values of ED_50_ and b (E_max_) were projected by yielding a minimum residual sum of squares of deviations from the theoretical curve^57^.

There were no missing data and all data were summarized and expressed as means ± SEM or 95% confidence limits. The statistical significance was evaluated by two-tailed and unpaired Student t-test and one-way or repeated measures two-way analysis of variance (ANOVA) using the 5.01 version of Prism (GraphPad Software, San Diego, CA, USA). The post-hoc Student-Newman-Keuls test was conducted when the effect of the drug (dose) (for the one-way ANOVA, the factor was drug [dose]; for the two-way ANOVA, the factors were drug [dose], time and their interaction) was observed to be statistically significant. The probability values were two-tailed and the statistical significance criterion P value was 0.05.

## Additional Information

**How to cite this article:** Wang, Y.-X. *et al*. Dezocine exhibits antihypersensitivity activities in neuropathy through spinal µ-opioid receptor activation and norepinephrine reuptake inhibition. *Sci. Rep.* **7**, 43137; doi: 10.1038/srep43137 (2017).

**Publisher's note:** Springer Nature remains neutral with regard to jurisdictional claims in published maps and institutional affiliations.

## Figures and Tables

**Figure 1:**
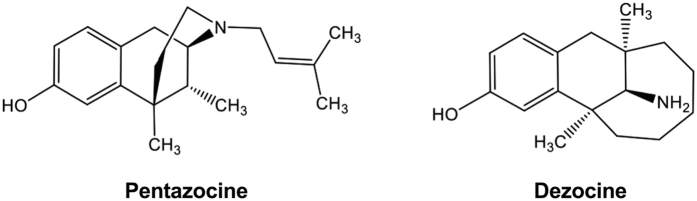
The chemical structures of pentazocine and dezocine.
